# MIMIC-IV, a freely accessible electronic health record dataset

**DOI:** 10.1038/s41597-022-01899-x

**Published:** 2023-01-03

**Authors:** Alistair E. W. Johnson, Lucas Bulgarelli, Lu Shen, Alvin Gayles, Ayad Shammout, Steven Horng, Tom J. Pollard, Sicheng Hao, Benjamin Moody, Brian Gow, Li-wei H. Lehman, Leo A. Celi, Roger G. Mark

**Affiliations:** 1 Massachusetts Institute of Technology, Cambridge, MA USA; 2 The Hospital for Sick Children, Toronto, ON Canada; 3 Beth Israel Deaconess Medical Center, Boston, MA USA

**Keywords:** Health services, Epidemiology, Public health

## Abstract

Digital data collection during routine clinical practice is now ubiquitous within hospitals. The data contains valuable information on the care of patients and their response to treatments, offering exciting opportunities for research. Typically, data are stored within archival systems that are not intended to support research. These systems are often inaccessible to researchers and structured for optimal storage, rather than interpretability and analysis. Here we present MIMIC-IV, a publicly available database sourced from the electronic health record of the Beth Israel Deaconess Medical Center. Information available includes patient measurements, orders, diagnoses, procedures, treatments, and deidentified free-text clinical notes. MIMIC-IV is intended to support a wide array of research studies and educational material, helping to reduce barriers to conducting clinical research.

## Abstract (summary)

**Table:** 

Measurement(s)	Homo sapiens
Technology Type(s)	Electronic Health Record
Sample Characteristic - Organism	Homo sapiens
Sample Characteristic - Environment	hospital
Sample Characteristic - Location	Commonwealth of Massachusetts

## Background

Thanks to the widespread adoption of electronic health record systems, data collected during routine clinical practice is now digitally stored in hospitals across the United States. Despite widespread storage of this data, archiving systems are often not designed to support research, making them difficult to navigate and access. In addition, routinely collected clinical data is often sporadic and noisy, reflecting the processes by which it was generated, where quality of data collection is understandably peripheral to the act of providing high quality care.

The intensive care unit (ICU) is an especially data-rich environment as patients require close monitoring. The typically acute nature of ICU patient illness and the importance of immediate intervention also make the environment of high-interest to researchers. Uniquely, there are a number of publicly available critical care datasets which have enabled research in this area. These projects largely build upon MIMIC, a waveform database with demographics digitally transcribed from paper records for over 90 patients^1^. MIMIC-II followed with a significantly increased sample size and breadth of information due to the clinical information being entirely sourced from various digital information systems^2^. More recently, MIMIC-III was published in 2015 and significantly expanded MIMIC-II, containing data for over 40,000 patients^3^. Outside of the MIMIC projects, a number of other critical care datasets have been made available to the worldwide research community. The eICU Collaborative Research Database (eICU-CRD) v2.0 comprises of 200,859 stays at ICUs and step-down units across 208 hospitals in the continental United States^4^. The AmsterdamUMCdb provides granular information for 23,106 admissions of 20,109 unique individuals admitted to a single academic medical center in the Netherlands^5^. The HiRID database contains high-resolution data for almost 34,000 admissions between 2008–2016 at Bern University Hospital in Switzerland^6,7^. HiRID contains 712 routinely collected physiological variables with one data entry every two minutes. The Pediatric Intensive Care (PIC) database is sourced from The Children’s Hospital at Zhejiang University School of Medicine with 12,881 patients and 13,941 ICU stays admitted from 2010–2018^8^.

Although the increasing number of datasets publicly available for research is encouraging, a number of areas for improvement remain. Data content varies considerably across datasets, with each having a particular strength. HiRID contains high resolution physiologic variables, eICU-CRD spans hundreds of distinct hospitals, while PIC contains pediatric patients. Clinical practice evolves quickly, requiring continual updating of the resources in order for derivative research to remain relevant. Finally, most datasets comprise of only one modality of information, clinical observations, and omit other important domains such as imaging, free-text, physiologic waveforms, and genomics.

In this paper we describe the public release of MIMIC-IV, a contemporary electronic health record dataset covering a decade of admissions between 2008 and 2019. MIMIC-IV complements the growing area of publicly accessible critical care datasets in a number of ways. First, MIMIC-IV is contemporary, containing information from 2008–2019. Second, MIMIC-IV incorporates new precise digital information sources such as the electronic medicine administration record. Third, MIMIC-IV establishes a modular organization of the constituent data allowing linking of the database to external departments and distinct modalities of data.

## Methods

**Fig. 1:**
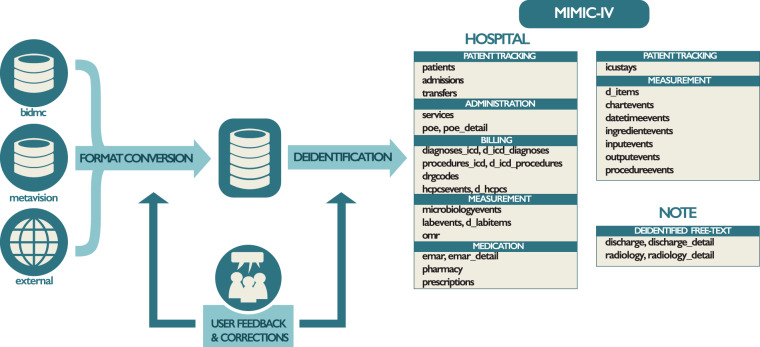
An overview of the development process for MIMIC. Data are acquired from the BIDMC data warehouse, the ICU information system (MetaVision), and external sources (“acquisition”). Structured Query Language (SQL) scripts merge the various data sources into a single schema (“transformation”). Finally, deidentification algorithms are applied to selectively remove protected health information from the reformatted schema. Tables present in MIMIC-IV are provided on the far right of the figure under their respective module. Issues raised on the MIMIC Code Repository are assessed and used to improve the build process as appropriate.

MIMIC-IV is the result of a collaboration between Beth Israel Deaconess Medical Center (BIDMC) and Massachusetts Institute of Technology (MIT). Data collected at BIDMC as part of routine clinical care is deidentified, transformed, and made available to researchers who have completed training in human research and signed a data use agreement. The Institutional Review Board at the BIDMC granted a waiver of informed consent and approved the sharing of the research resource. Broadly, the creation of MIMIC involved three distinct steps: acquisition, transformation, and deidentification. Figure 1 gives an overview of the data acquisition, transformation, and deidentification process.

### Acquisition

The majority of data in MIMIC-IV is generated and archived within BIDMC as part of routine clinical care and related activities such as monitoring, provider orders, and billing. This data is initially brought together in a Microsoft SQL Server Database within a secure data warehouse at the hospital. Subsequently, the data is transferred to a PostgreSQL database system (PostgreSQL 12.8, PostgreSQL Global Development Group) on MIT secure servers via a Virtual Private Network (VPN) connection. Complementary datasets such as code system definitions (e.g. International Classification of Diseases (ICD)) and state death records are acquired independently of BIDMC and loaded onto the PostgreSQL database located at MIT.

**Fig. 2:**
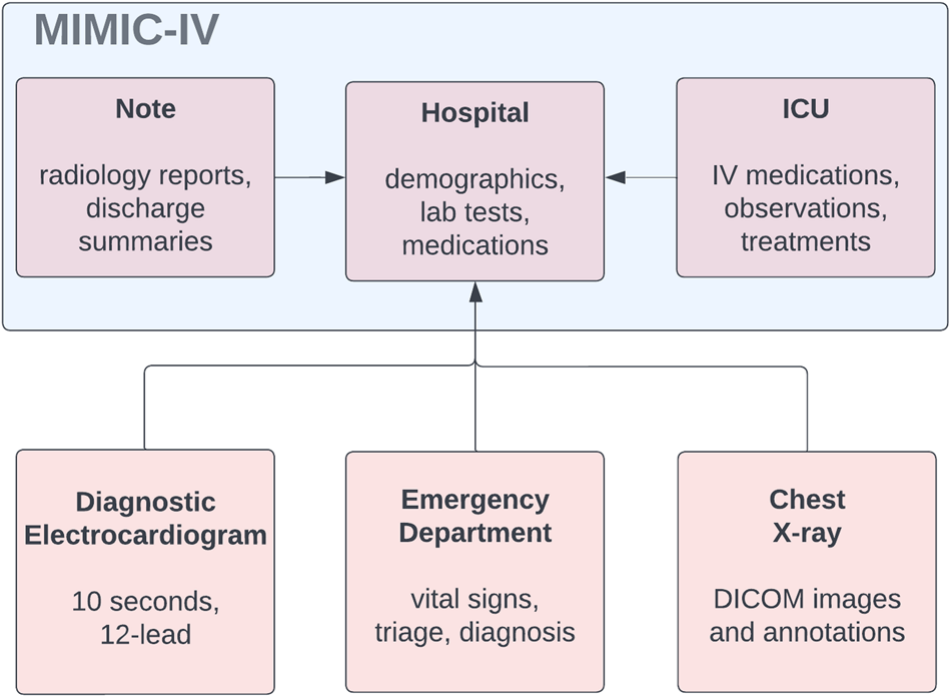
MIMIC-IV follows a modular structure. Modules can be linked by identifiers including subject_id, hadm_id, and deidentified date and time. Example content of each module is shown.

#### Clinical cohort

We acquired information from BIDMC for all patients who were admitted to either the emergency department or an ICU between 2008–2019. Patients were excluded if they were younger than 18 on their first visit or if they were on a known list of individuals requiring enhanced protection. A small number of medical record numbers corresponding to the same individual were merged.

For extracting EHR data from BIDMC, we accessed the comprehensive data warehouse, mentioned previously, as well as a MetaVision (iMDsoft, Israel) bedside clinical information system used in the critical care units. Some data elements are duplicated between the two systems because the hospital-wide EHR pushes information into MetaVision (e.g. an HL7 integration engine automatically synchronizes laboratory results). Data was transferred to MIT using the PGLoader export tool^9^.

#### External data sources

Clinical data often require external data sources, such as coding systems, for intepretation. Examples systems include Diagnosis Related Groups (DRGs), the International Classification of Diseases (ICD), and the Healthcare Common Procedure Coding System (HCPCS). We acquired these reference systems from publicly available sources and included them in the database to facilitate analysis. For DRG, we loaded two versions of the coding system: All Payer (AP-DRG) and Medicare Severity (MS-DRG). Descriptions for AP-DRGs were acquired from the MassHealth publication of DRGs and related costs^10^. Descriptions for MS-DRGs were acquired from the Healthcare Cost and Utilization Project (HCUP) tools^10^.

ICD codes were acquired for both version 9 and 10 of the coding system. Codes were acquired from the Centers for Medicare & Medicaid Services (CMS)^11,12^. The ICD coding systems are updated yearly, with codes being added, removed, or updated as necessary. As MIMIC-IV has been collected over the span of a decade, a number of codes used historically have since been removed from the latest version. To allow for interpretation of these historical codes, we initialized the ICD dimension table with the respective 2020 version. We then sequentially added codes no longer present by iterating over past versions. At the end of the creation of MIMIC-IV, we verified that all ICD codes in MIMIC-IV were described in the dataset. Note that we conducted this sequential process for all versions and systems of ICD in MIMIC-IV, namely: ICD-9-CM, ICD-9-PCS, ICD-10-CM, and ICD-10-PCS.

#### Out-of-hospital mortality

Due to data quality concerns regarding the Social Security Death Master File (SSDMF)^13^, we did not attempt to link patients to the SSDMF for determining out-of-hospital mortality. Instead, we linked patient records to the Massachusetts State Registry of Vital Records and Statistics^14^. We created a custom algorithm which matched records using name, social security number, and date of birth using the RecordLinkage library v0.15^15^.

Our matching algorithm first generated a list of candidate matches by requiring at least one exact match for the following three feature combinations: first and last name, social security number, or last name and date of birth. We then used fuzzy matching approaches to rank the quality of the match. Exact matches were ranked highest, followed by approximate matches using either the Jaro-Winkler edit distance for names, Levenshtein edit distance for social security numbers, or a date-specific edit distance for dates. After ranking, we iterated through a series of custom rules for matching until only unlikely matches remained. These custom rules set the minimum distance allowable for pairing two records as well as the order of identifiers used for linking. We aimed to maximize sensitivity in the linkage process.

Of the original 590,325 unique individuals in the BIDMC EHR, our initial list of candidates resulted in 717,092 links for 150,832 unique individuals, i.e. 25.6% of individuals had a potential match. We ultimately linked 65,805 of the 150,832 unique individuals (43.6%) to state death records. The majority of links were made using an exact match on last name, date of birth, and social security number (51,022/65,803, 77.5%). The second highest number of matches were made using an exact match on last name and date of birth and an approximate match on first name (11,634/65,803, 17.7%). The remaining matches were evenly distributed among the other rules which typically required an exact match on one high cardinality identifier (such as last name and date of birth) and an approximate match on another identifier.

### Transformation

Custom Structured Query Language (SQL) scripts were used to transform the data to the final MIMIC structure. Two principles were adhered to during this process. First, we aimed to maintain backward compatibility with MIMIC-III. Second, we sought to minimize processing to align the published data as closely to the data recorded during clinical practice.

MIMIC-IV adopts a relational structure with predefined relationships stored across tables and columns. Data are grouped into three modules: **hosp**, **icu**, and **note**. These modules serve a similar purpose to schemas in database organization in that they support human interpretation of the underlying data structure. Conceptually, a module contains a subset of information which has similar provenance or content Fig. 2. Modules in MIMIC-IV include **hosp**, **icu**, and **note**. The modular structure is intended to enable future linkage of MIMIC-IV with data sourced from other departments and with varying modalities of information.

Admission/discharge/transfer (ADT) records are placed in the **hosp** module, alongside other hospital-wide data such as laboratory values, microbiology cultures, medication orders, and administrative data derived from hospital billing practices. Note that while the majority of information is collected at the BIDMC, some information was transferred to the BIDMC from outpatient clinics.

The **icu** module comprises data documented at the ICU bedside. Data includes intravenous infusions, patient outputs, charted observations, and documentation of ongoing procedures. The **note** module comprises discharge summaries and radiology reports. To support pairing of free-text notes with structured information, we created a single entity-attribute-value table for each note type. For example, the *radiology_detail* table contains quantitative information typically collected with radiology reports.

After creation, tables were exported to comma separated value (CSV) files following the RFC-4180 memo to facilitate sharing and reuse^16^.

### Deidentification

The Health Insurance Portability and Accountability Act (HIPAA) Safe Harbor provision stipulates a set of 18 identifiers (for example, names, locations, serial numbers, and ages) which must be removed in order for a dataset to be considered deidentified. Once deidentified, data may be shared beyond the covered entities involved in initial collection and processing. We developed custom algorithms to find and remove HIPAA identifiers.

Look-up tables were used to randomly assign patients with a unique identifier (subject_id) and hospitalizations with a unique identifier (hadm_id). Dates were perturbed by shifting them using a patient-level offset. The shift ensures that the interval between two time points for a patient is preserved.

Finally, we combined two published algorithms for removing PHI from free-text^17,18^. If either of these algorithms identified an entity as PHI, it was removed from the database and replaced with three consecutive underscores (“___”). We applied this approach to the free-text clinical notes in the **note** module as well as free-text elements present in the structured data (e.g. comment fields). We validated our deidentification approach with thorough human review and, where possible, created exhaustive allow lists which filtered data prior to release.

## Data Records

Access to MIMIC-IV is provided via PhysioNet^19^. The **hosp** and **icu** modules in MIMIC-IV are available in the MIMIC-IV project on PhysioNet^20^. The **note** module is available from the MIMIC-IV-Note: Deidentified free-text clinical notes project on PhysioNet^21^.

**Fig. 3:**
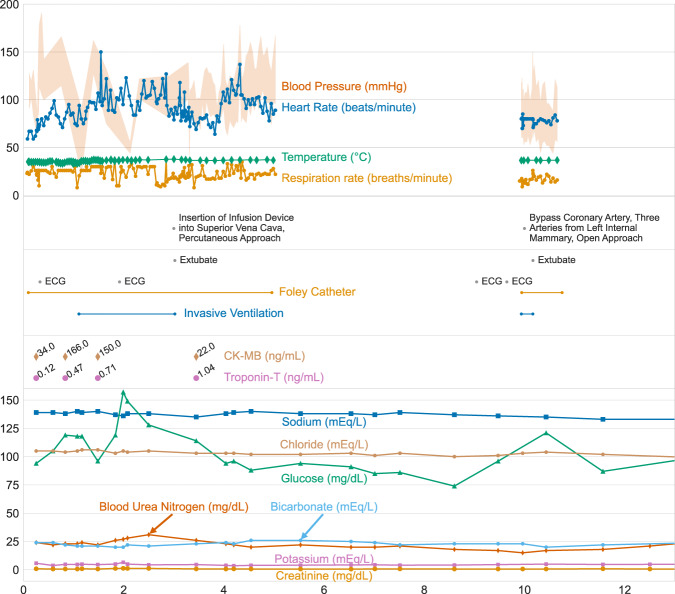
Visualization of data within MIMIC-IV for a single patient’s hospitalization: hadm_id 28503629. Three vertically stacked panels highlight the variety of information available. Vital signs are shown in the top panel: note the frequency of data collection for temperature is much higher at the start of the ICU stay due to the use of targeted temperature management. Procedures from multiple sources are shown in the middle panel, including from billing information, the provider order entry system, as well as the ICU information system. The bottom panel displays patient laboratory measurements. Note that while frequent vital signs are only available when the patient is in the ICU, laboratory measures are available throughout their hospitalization.

Table 1 summarizes the demographics for ICU patients in MIMIC-IV. Figure 3 visualizes a patient who is admitted to an ICU for a cardiac arrest, discharged to a general ward, admitted to the operating room, has a planned readmission to an ICU after their operation, and is ultimately discharged home.

**Table 1:** Demographics for patients admitted to an intensive care unit (ICU) in MIMIC-IV v2.2.

	Hospital admissions	ICU admissions
Number of stays	431,231	73,181
Unique patients	180,733	50,920
Age, mean (SD)	58.8 (19.2)	64.7 (16.9)
Female Administrative Gender, n (%)	224,990 (52.2)	32,363 (44.2)
Insurance, n (%)		
Medicaid	41,330 (9.6)	5,528 (7.6)
Medicare	160,560 (37.2)	33,091 (45.2)
Other	229,341 (53.2)	34,562 (47.2)
Hospital length of stay, mean (SD)	4.5 (6.6)	11.0 (13.3)
In-hospital mortality, n (%)	8,974 (2.1)	8,519 (11.6)
One year mortality, n (%)	106,218 (24.6)	28,274 (38.6)

### Hospital module (hosp)

The **hosp** module stores information regarding patient transfers, billed events, medication prescription, medication administration, laboratory values, microbiology measurements, and provider orders. The subject_id column is present in all tables and allows linkage to patient demographics in the *patients* table. The hadm_id column is also present in all tables and represents a single hospitalization; rows without an hadm_id pertain to data collected outside of an inpatient encounter. Most tables may be interpreted without cross-linking to other tables. Tables which contain item_ids are an exception, and they must be linked to a dimension table prefixed with *d_* in order to acquire a human interpretable description of the item_ids. Other tables, such as *emar* and *poe*, may be linked with “detail” tables (*emar_detail* and *poe_detail*) which provide additional information for each row.

#### Patient tracking

Patient demographics and in-hospital movement are described in three tables: *patients*, *admissions*, and *transfers*. Each distinct patient is assigned a subject_id, and the *patients* table has a unique *subject_id* for each row. The *patients* table provides the patient’s administrative gender, their age, and their date of death.

In order to appropriately deidentify the exact date of patient stays, the *patients* table contains an anchor_year column. This column “anchors” data stored in the *patients* table to a year occurring in their deidentified timeline (e.g. 2150). At this deidentified year, the patient’s age is provided in the anchor_age column and the approximate true year of admission is provided in the anchor_year_group column. For example, if a patient’s anchor_age is 50 and their anchor_year is 2150, then they were 50 years old in the year 2150. Continuing the example, if this patient’s anchor_year_group is 2011–2013, then we know that any hospitalizations occurring in the deidentified year 2150 (i.e. the anchor_year) actually occurred sometime between 2011 and 2013, and that they were 50 years old during this time period. The anchor_year_group column was added to MIMIC-IV to allow analyses which incorporate changes in medical practice over time.

Finally, a patient’s date of death is available in the dod column. Dates of death are censored at one-year from the patient’s last hospital discharge. As a result, null dates of death indicate the patient was alive at least up to that time point. Inferences regarding patient death beyond one year cannot be made using MIMIC-IV. The majority of patient death information is acquired from hospital records when the individual dies within the BIDMC or an affiliated institute.

#### Administration

Three tables in the **hosp** module provide administration related information: *services*, *poe*, and *poe_detail*. The *services* table provides information on the hospital-related service under which a patient is hospitalized. The *poe* and *poe_detail* tables contain orders made in the provider order entry (POE) system. The POE system is used within the hospital to make orders related to diagnoses, imaging, consultation, and treatment. Typically the *poe* tables provide the date and time of an order such as an x-ray study, medication order, or nutrition order, but provide limited detail about the order itself.

#### Billing

Billing information is stored in the *diagnoses_icd*, *procedures_icd*, *drgcodes*, and *hcpcsevents* tables. The *diagnoses_icd* table contains coded diagnoses representing the hospitalization as determined by trained professionals after reviewing signed patient notes. The ontology of the *diagnoses_icd* table is the International Classification of Diseases, Ninth Revision, Clinical Modification (ICD-9-CM) diagnoses and the ICD Tenth Revision, Clinical Modification (ICD-10-CM) diagnoses. Definitions for ICD codes are provided in the *d_icd_diagnoses* table. A maximum of 39 diagnoses may be billed for a single hospital encounter, and seq_num provides an approximate ordering of diagnosis. There are few incentives for the billing department to ensure seq_num is a perfect rank ordering of diagnosis importance, however, and caution should be taken when using seq_num for research purposes. A similar table structure is adopted for billed procedures which are stored in the *procedures_icd* table with descriptions of codes provided in the *d_icd_procedures* table.

Diagnoses are recorded with the ICD-9-CM or ICD-10-CM ontologies, while procedures are recorded with the ICD-9-PCS or ICD-10-PCS ontologies. As these ontologies were updated throughout the data collection period of MIMIC-IV, the *d_icd_diagnoses* and *d_icd_procedures* tables contain all codes which were valid at any point during the 2008–2019 time period.

Diagnosis Related Groups (DRGs) are billable codes used to assign an overall cost to a hospitalization. Many ontologies for DRG codes exist, and the drg_type column stores the ontology for the given row. The final billing tables are *hcpcsevents* and its associated dimension table *d_hcpcsevents*. The *hcpcsevents* table records billing by the hospital for provided services such as mechanical ventilation or provision of ICU care.

#### Measurement

Measurements sourced from patient derived specimens are available in *microbiologyevents* and *labevents*, with the *d_labitems* table providing definitions for concepts present in the *labevents* table. Laboratory measurements have a simpler structure compared to microbiology measurements though both relate to patient derived specimens such as blood. Multiple measurements are often taken for a single specimen, delineated by the specimen_id column in the *labevents* table and the micro_specimen_id column in *microbiologyevents*. For example, blood gas measurements made on the same sample will share the same specimen_id with one concept specifying the type of specimen (arterial, venous, etc).

Microbiology measurements are stored in a single table with columns dedicated to domain specific concepts. Measurements follow a directed hierarchy of specimen, organism, isolate, antibiotic, and dilution. To simplify analysis of this data, elements higher in the hierarchy such as specimen and organism are repeated for elements lower in the hierarchy such as antibiotic and dilution. Microbiology cultures typically have interim results reported to the care providers. This information is not captured in this table, which only provides the final interpretation of a microbiology culture as it was documented at storetime.

Finally, the *omr* table provides information from the Online Medical Record (OMR) for the patient. OMR is a general system used for documenting patient information from visits at BIDMC affiliated institutes. As of MIMIC-IV v2.2, the OMR table contains data for five measurements: blood pressure, height, weight, body mass index, and the Estimated Glomerular Filtration Rate (eGFR). These values are available from both inpatient and outpatient visits, and in many cases a “baseline” value from before a patient’s hospitalization is available.

#### Medication

There are four tables in the **hosp** module which track medication prescription and administration: *prescriptions*, *pharmacy*, *emar*, and *emar_detail*. The *prescriptions* and *pharmacy* tables are intended to be used together: *prescriptions* contains the order made by a provider and *pharmacy* stores detailed information regarding the compound prescribed. Not all prescribed compounds are associated with an entry in the *pharmacy* table.

The other two medication related tables, *emar* and *emar_detail*, are sourced from the electronic Medicine Administration Record (eMAR). The eMAR system requires barcode scanning of a patient wristband and the medication at the time of administration and was deployed throughout the BIDMC between 2014–2016. By 2016, all units of the hospital had the eMAR system deployed, and thus all hospitalizations from 2016 onward would be anticipated to have records within eMAR. Unlike the *prescriptions* table which stores medication requests, the eMAR system records administration. The *emar* table has one row per administration, with emar_id uniquely identifying rows and emar_seq being a monotonically increasing integer ordering events chronologically. The poe_id and pharmacy_id columns allow linking to the *poe* and *pharmacy* tables, respectively. Importantly, every row in *emar* links to one or more rows in *emar_detail*. As each formulary dose must be scanned as a part of the workflow, an administration of 200 mg with formulary doses of 100 mg will result in three rows in emar_detail: one row for the overall administration (with a missing value for parent_field_ordinal), and two rows for each scanned formulary dose (with increasing values of parent_field_ordinal). Columns which describe the entire administration event such as complete_dose_not_given and dose_due are only present for the primary row. Most columns refer to individual formulary doses (dose_given, product_description, and so on), and are only present for the formulary dose rows.

**Fig. 4:**
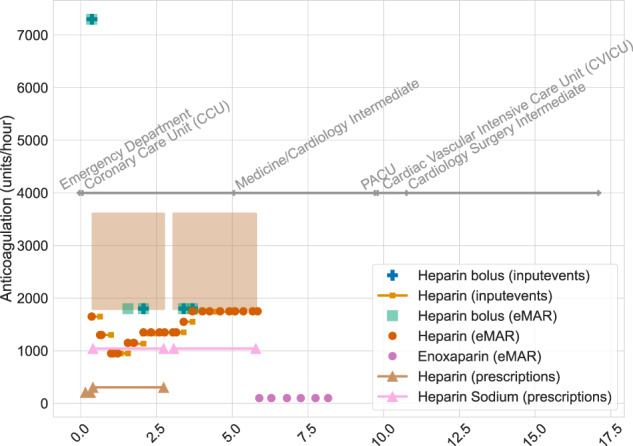
Visualization of medication information documented within MIMIC-IV for a single patient’s hospitalization: hadm_id 28503629. The annotated grey line indicates care units for the patient throughout their stay. Bolus medications are indicated by markers, continuous infusions as lines, and range doses as filled boxes. For example, on day 5 of their hospital stay, the patient had two active prescriptions for heparin (one for 1600–3500 units of heparin, brown filled box, and one for 1000 units of heparin, pink line with triangles). Additionally on day 5, the patient continued to received heparin according to *emar* (orange circle), and was imminently transferred to the medicine/cardiology intermediate ward.

Figure 4 visualizes the complementary information present in the *emar*, *emar_detail*, *prescriptions*, and *inputevents* tables for a single patient.

### ICU module (icu)

The MetaVision clinical information system (iMDsoft, Israel) is the source of data for patients admitted to the ICU. MetaVision was the only clinical information system used in the ICU for the time period of data collection for MIMIC-IV. Tables in the **icu** module include *chartevents*, *d_items*, *datetimeevents*, *icustays*, *inputevents*, *outputevents*, and *procedureevents*. The **icu** module adopts a star schema, with all event tables referencing *d_items* for defining itemid and *icustays* for defining stay_id.

The stay_id column is a primary key for the *icustays* table, and as such is unique for each row. ICU stays are defined using the administrative record of patient movement within the hospital, i.e. the *icustays* table is derived from the *transfers* table in the **hosp** module. ICU stays are identified using a lookup table matching physical location to an ICU cost center. Each transfer that corresponds to an ICU stay is assigned a stay_id, and consecutive transfers are merged into a single stay_id. The time of admission (intime) and discharge (outtime) are available in the *icustays* table for each ICU stay. Importantly, if a transfer to a non-ICU ward occurs between two ICU stays, a unique stay_id will be assigned to each of the two stays.

Each documented item is colloquially referred to as an “event” in the **icu** module, and events are grouped into tables based on the underlying data type. Events which correspond to dates, such as the time of last dialysis, are stored in *datetimeevents* with the value column corresponding to the date. Continuous and bolus infusions are provided in the *inputevents* table with a starttime, endtime, rate, and amount. Patient outputs are documented in the *outputevents* table with a single numeric value occurring at a single charttime. The *procedureevents* table captures processes which have a starttime and endtime including organ support treatments such as mechanical ventilation. Finally, the *chartevents* table is the largest of all the events table, and acts as a catch-all for documentation at the bedside. Each row in *chartevents* has a charttime, indicating the time at which the measurement was relevant and a value column storing the value documented. All events tables contain patient subject_id, hadm_id, and stay_id, as well as a storetime indicating the time at which the measurement was validated by bedside staff.

### Notes module (note)

The **note** module contains free-text, deidentified clinical notes. The notes are organized into two tables: *discharge*, and *radiology*. Discharge summaries, stored in the *discharge* table, are in-depth notes which overview a patient’s history and course throughout a given hospitalization. Discharge summaries are organized into sections including chief complaint, history of present illness, past medical history, brief hospital course, physical exams, and discharge diagnoses. As a part of the deidentification process, the Social History and Discharge Instructions sections have been removed. These sections typically contained social and logistical information which was irrelevant for medical care but introduced a higher risk of reidentification as compared to other sections. Auxiliary information associated with each note has been stored in entity-attribute-value tables with the “_detail” suffix. For the discharge summaries these data are available in the *discharge_detail* table.

The *radiology* table contains radiologist reports for imaging studies performed. Radiology reports cover a wide set of imaging modalities including x-ray, computed tomography, magnetic resonance imaging (MRI), and ultrasound. Radiology reports follow structured reporting best practices and have a dedicated section for the indication, comparison, findings, and impression of the imaging study. For more in-depth imaging scans such as full body MRIs, reports may describe findings organized according to the body system examined. The associated *radiology_detail* table provides a coded ontology for radiology examinations as well as current procedural terminology (CPT) codes for each study. If an addendum for a report exists, the *radiology_detail* provides the associated note_id.

### Building on earlier versions of MIMIC

MIMIC-IV is similar in many ways to previous versions of MIMIC. Tables with identical names in MIMIC-III and MIMIC-IV will be broadly compatible. The similarities help to ensure that code and analyses can be carried over from studies developed on earlier versions of the database. Table 2 summarizes notable changes for users transitioning from MIMIC-III to MIMIC-IV.

**Table 2:** Major changes between MIMIC-III v1.4 and MIMIC-IV v2.2.

MIMIC-IV table	Description of change
*emar*, *emar_detail*, *ingredientevents*, *omr*, *poe*, *poe_detail*, *pharmacy*	New tables only present in MIMIC-IV.
*labevents*	Added the following columns: storetime, specimen_id, ref_range_lower, ref_range_upper, priority, and comments.
*microbiologyevents*	Added the following columns: micro_specimen_id, test_seq, storedate, storetime, test_name, test_name, quantity, comments.
*labevents*	Added the following columns: storetime, specimen_id, ref_range_lower, ref_range_upper, priority, and comments.
*hcpcsevents*, *d_hcpcs*	Replaced the *cptevents* and *d_cpt* tables.
*prescriptions*	Columns starttime and endtime replaced startdate and enddate as the associated time is now available.
	Columns drug_name_generic and drug_name_poe were removed.
	Columns pharmacy_id, form_rx, and pharmacy_id were added.
*inputevents*	Renamed; equivalent to *inputevents_mv* in MIMIC-III. *inputevents_cv* has been removed.
*procedureevents*	Renamed; equivalent to *procedureevents_mv* in MIMIC-III.
*icustays*	The unit-level identifier *icustay_id* has been replaced with the general location-based identifier *stay_id*. *stay_id* is used to identify a period of stay within a single location.

Importantly, all patient identifiers have been regenerated for MIMIC-IV. As a result, it is not possible to link patients across the databases using an identifier such as subject_id, even though MIMIC-III and MIMIC-IV have an overlap in their data collection periods (specifically the years 2008–2012). To support research that spans the periods of MIMIC-III (2002–2008) and MIMIC-IV (2008–2019), we have published the MIMIC-III Clinical Database CareVue subset^22^. The CareVue subset contains only those patients from MIMIC-III who are not in MIMIC-IV.

## Technical Validation

The creation of MIMIC-IV followed best practices in scientific computing^23^. An interdisciplinary team of scientists and clinicians evaluated MIMIC-IV during development, conducted code reviews, and tracked issues using a ticket system. Code was managed using version control software. Data transformation was executed with a single, reproducible build script. Validation of the build process assessed data integrity, data consistency, and deidentification. Unit tests were created to apply these checks during development.

Integrity checks were used to ensure integrity of the database structure. For example, a simple unit test would verify that each diagnosis code in *diagnoses_icd* was present in the associated dimension table, *d_icd_diagnoses*. Another check would ensure that every table containing patient data had an associated entry in the *patients* table. A similar integrity check was performed for tables with an hadm_id column using the *admissions* table.

Consistency checks were used to confirm that information within the data was consistent with the known truth. For example, almost all ICU stays should have a billed diagnosis. Our testing framework therefore required a minimum of 99% of hadm_id in the *icustays* table to be present in the *diagnoses_icd* table. Similarly, we verified that 99% of ICU stays in the *icustays* table had at least one heart rate measurement in the *chartevents* table. We also ran comparisons of MIMIC-IV against earlier versions of MIMIC (e.g. we verified a subset of itemid in the *labevents* table matched the values in MIMIC-III v1.4).

Deidentification checks were used to confirm that the deidentification pipeline had been successfully applied to the dataset. For example, tests would extract all unique values for a column in a table and verify that every value was present in a manually curated allow list. Other checks sought to verify that dates were perturbed and that sensitive attributes such as race had been sufficiently aggregated.

## Usage Notes

MIMIC-IV contains a vast amount of information about clinical care. As with previous versions of the database, we anticipate a broad range of downstream uses including research, education, and technology development.

### Access

Individuals interested in accessing MIMIC-IV must complete a training course in research with human participants and sign a data use agreement (DUA). The DUA requires users to adequately safeguard the dataset, to not attempt to reidentify individuals, to not share the data, and to report issues relating to deidentification.

### Derived tables

The MIMIC Code Repository was originally created for MIMIC-III to enable researchers to share and reuse code^24^. For example, the repository includes SQL scripts to extract clinically meaningful subsets of information such as blood gases, ventilation settings, and vasopressor treatment. We will continue to use of the MIMIC Code Repository for MIMIC-IV^25^. Scripts to load MIMIC-IV data into a number of relational database management systems (e.g. PostgreSQL, MySQL, and Google BigQuery) have already been made available. In addition, much of the existing MIMIC-III code has been migrated to MIMIC-IV.

Where possible, we have made MIMIC-IV and the reorganized data generated by code in the MIMIC Code Repository directly available for interrogation on the cloud. We have also shared example analyses and tutorials as executable documents (e.g. Jupyter Notebooks, RMarkdown). Separate to the MIMIC Code Repository, we have released source code that fully reproduces a published study using MIMIC-IV and we hope this serves as a template for future research^26^.

## Data Availability

The entire build process cannot be made publicly available due to the inclusion of sensitive patient information. Our deidentification approach combined rule based approaches with a neural network. The rule based approach is published and publicly available^27^. The neural network is also publicly available and has been described elsewhere^18^. Online documentation describing the database in detail is available online at https://mimic.mit.edu. The source code to generate this documentation is open for contributions^28^.
