# Author Correction: MIMIC-IV, a freely accessible electronic health record dataset

**DOI:** 10.1038/s41597-023-01945-2

**Published:** 2023-01-16

**Authors:** Alistair E. W. Johnson, Lucas Bulgarelli, Lu Shen, Alvin Gayles, Ayad Shammout, Steven Horng, Tom J. Pollard, Benjamin Moody, Brian Gow, Li-wei H. Lehman, Leo A. Celi, Roger G. Mark

**Affiliations:** 1 Massachusetts Institute of Technology, Cambridge, MA USA; 2 The Hospital for Sick Children, Toronto, ON Canada; 3 Beth Israel Deaconess Medical Center, Boston, MA USA

**Keywords:** Health services, Epidemiology, Public health

Correction to: *Scientific Data* 10.1038/s41597-022-01899-x, published online 3 January 2023.

Two column headings, “Hospital admissions” and “ICU admissions”, were missing from Table 1 in the original version of the paper. These have been corrected in the HTML and pdf versions.

