# Author Correction: MIMIC-IV, a freely accessible electronic health record dataset

**DOI:** 10.1038/s41597-023-02136-9

**Published:** 2023-04-18

**Authors:** Alistair E. W. Johnson, Lucas Bulgarelli, Lu Shen, Alvin Gayles, Ayad Shammout, Steven Horng, Tom J. Pollard, Sicheng Hao, Benjamin Moody, Brian Gow, Li-wei H. Lehman, Leo A. Celi, Roger G. Mark

**Affiliations:** 1 Massachusetts Institute of Technology, Cambridge, MA USA; 2 The Hospital for Sick Children, Toronto, ON Canada; 3 Beth Israel Deaconess Medical Center, Boston, MA USA

**Keywords:** Health services, Epidemiology, Public health

Correction to: *Scientific Data* 10.1038/s41597-022-01899-x, published online 3 January 2023.

An author of the paper was omitted in the original version (Sicheng Hao, Massachusetts Institute of Technology, USA). This has been corrected in the pdf and HTML versions of the paper, and the associated metadata.

